# Anxiety, depression, and brain overwork in the general population of Mongolia

**DOI:** 10.1038/s41598-024-52779-w

**Published:** 2024-01-30

**Authors:** Battuvshin Lkhagvasuren, Tetsuya Hiramoto, Enkhjin Bat-Erdene, Enkhnaran Tumurbaatar, Gantsetseg Tumur-Ochir, Tsolmontuya Amartuvshin, Myagmartseren Dashtseren, Edward Lai, Vijay Viswanath, Takakazu Oka, Tsolmon Jadamba

**Affiliations:** 1 Brain and Mind Research Institute, Mongolian Academy of Sciences, Ulaanbaatar, Mongolia; 2 Department of Psychosomatic Medicine, International University of Health and Welfare Narita Hospital, Narita, Japan; 3 Department of Psychosomatic Medicine, NHO Fukuoka National Hospital, Fukuoka, Japan; 4 Department of Neuroscience and Cell Biology, Robert Wood Johnson Medical School, Child Health Institute of New Jersey, Rutgers University, New Brunswick, USA; 5 Department of Mental Health Surveillance, National Center for Mental Health, Ulaanbaatar, Mongolia; 6 Department of Family Medicine, School of Medicine, Mongolian National University of Medical Sciences, Ulaanbaatar, Mongolia; 7 School of Osteopathic Medicine, Rowan-Virtua University, Stratford, USA; 8 College of Medicine, University of Cincinnati, Cincinnati, USA

**Keywords:** Epidemiology, Human behaviour, Anxiety, Depression, Medical research, Risk factors

## Abstract

In Mongolia, there is limited data on the prevalence and correlates of common mental health conditions. This study addresses this data gap by exploring anxiety, depression, and brain overwork. The aim of this study was to determine normative data on these conditions in the general population of Mongolia. This nationwide, population-based, cross-sectional study was conducted in 48 sampling centers across Mongolia in 2020. A total of 613 participants (190 men and 423 women) with a mean age of 41.8 ± 12.4 years were recruited. The participants completed the Hospital Anxiety and Depression Scale (HADS) and the Brain Overwork Scale (BOS-10). Vital signs, body measurements, and lifestyle determinants were also assessed. The prevalence of anxiety was 9.9%, depression was 4.9%, and brain overwork was 18.3% among the participants. Anxiety and depression were correlated with brain overwork symptoms. Brain overwork was associated with young age, unemployment, low income, and alcohol use. These findings suggest that anxiety, depression, and brain overwork are a significant problem in the general population of Mongolia. Further research is needed to develop effective interventions to reduce the prevalence and risk factors of anxiety, depression, and brain overwork.

## Introduction

Mental disorders are a significant worldwide public health problem, with a high prevalence in many countries^1^. One in eight people in the world are affected by a mental disorder^2^. However, understanding the complexities of mental well-being requires a nuanced approach that avoids confusion between overlapping concepts. While mental disorders represent specific diagnoses with defined criteria, mental distress often serves as a precursor or even a symptom of these conditions. Mental distress can decrease quality of life, cause disability, and increase mortality. Consequently, it poses substantial challenges to the mental well-being of individuals^3^. Anxiety and depression, characterized by distinct emotional and cognitive symptoms, are undoubtedly central to mental well-being. Anxiety manifests as excessive worry, fear, and physical tension, while depression casts low mood, hopelessness, and diminished energy. However, the experience of mental distress often goes beyond these classic presentations. Individuals may exhibit somatic symptoms like fatigue, unexplained pain, or sleep disturbances, masking their underlying emotional burden^4^. These presentations often lead to diagnoses of medically unexplained symptoms or functional somatic syndromes, further complicating the understanding and treatment of their distress^5,6^. These patients often respond well to treatment with antidepressants and anxiolytics, suggesting that the cause of mental distress may be linked to chronic stress-induced brain activity dysfunction^7^. It is within this context that we propose the concept of brain overwork as a potential bridge between emotional and physical manifestations of mental distress. Drawing on Selye's General Adaptation Syndrome^8^, we hypothesize that chronic stress triggers an overactive stress response, leading to an "unrelenting workload" on the brain. This heightened activity, mediated by the hypothalamic–pituitary–adrenal axis and other stress-related pathways, may manifest in various ways, including: (i) excessive cognitive activity, characterized by constant rumination, intrusive thoughts, and difficulty "switching off.", (ii) emotional hypervigilance leading to heightened sensitivity to perceived threats and emotional reactivity, (iii) restless behavior, including physical agitation, difficulty relaxing, and sleep disturbances. These subjective experiences, collectively termed brain overwork syndrome, may not align with existing diagnoses of anxiety or depression but could represent an underlying contributor to these conditions. Furthermore, physical characteristics, such as age, sex, and obesity may influence individual vulnerability to stress and exacerbate its potential consequences, including both mental and physical health problems^9–11^. Chronic stress activates the sympathetic nervous system, leading to increased heart rate, blood pressure, and changes in body temperature^12–14^. Furthermore, anxiety and depression are associated with alterations in breathing patterns and oxygen consumption, potentially impacting oxygen saturation levels^15^. While a cross-sectional design cannot establish causal direction, examining these physiological markers may provide valuable insights for early identification and intervention. To investigate our hypothesis, we employed a combination of self-report questionnaires and objective physical assessments in a representative sample of the Mongolian general population.

Mongolia is a landlocked country in East Asia, located between Russia and China. The most recent study on the epidemiology of mental disorders in the general population of Mongolia was conducted between 1976 and 1984, and it indicated that a population-wide prevalence of mental disorders was 1.8%^16^. Since the democratic revolution in 1990, numerous factors such as the political transition from communism to democracy, rapid urbanization, air pollution, lifestyle changes, shifts in disease burden, and economic turbulence over the past three decades have likely significantly impacted the mental health of the Mongolian population. However, no studies have more recently assessed the mental well-being of the general population in Mongolia.

Several mental distress assessment instruments, such as the Generalized Anxiety Disorder-7, Beck Depression Inventory, and Hospital Anxiety and Depression Scale (HADS), have been investigated for their validity and reliability across different cultures. These questionnaires generally possess robust psychometric properties with a focus on emotional symptoms. Notably, the HADS is a concise self-report questionnaire used to assess health-related mental distress in both general and clinical populations^17^. It comprises of 14 Likert-scale items that represent two latent domains: anxiety and depression. In a previous study, we translated and evaluated the psychometric properties of the Mongolian version of the HADS in the general population, demonstrating good validity and reliability for assessing mental distress^18^.

To evaluate brain overwork, we utilized the Brain Overwork Scale (BOS-10), which we developed and validated for the Mongolian population. The BOS-10 assesses three core symptoms: excessive thinking, hypersensitivity, and restless behavior. The scale demonstrated excellent internal consistency (McDonald's ω = 0.861) and moderate external reliability (intraclass correlation coefficient = 0.75), indicating its accuracy and consistency in measuring brain overwork in this population. Moreover, the BOS-10's brevity and focus on specific symptoms not fully captured by other measures make it a valuable tool for research and clinical use^19^.

This study aimed to establish normative data and assess the screening ability of the HADS and BOS-10 for identifying anxiety, depression, and brain overwork in the general Mongolian population. Furthermore, we extensively investigated the sociodemographic and physical characteristics of the Mongolian population to determine factors associated with these conditions.

## Materials and methods

### Study participants

This cross-sectional study was part of a nationwide, multicenter, interdisciplinary, prospective, population-based cohort study that aimed to examine brain-related disorders in the general population of Mongolia. To ensure our study reflects the Mongolian population aged 18–65, we calculated an initial sample size of 385. This was determined using a 95% confidence level and a 5% margin of error, considering an estimated prevalence of 50% for the characteristic of interest and a population size of 2,028,035 individuals^20^. Taking into account the lifestyle differences between nomadic herders in rural areas and urban dwellers in urban areas, we included two residency locations, resulting in a target sample size of 770. Considering an 80% response rate, the final sample size required was 924. The inclusion criteria require: (i) participants be Mongolian citizens who have resided in geopolitical units for at least 6 months, (ii) be aged between 18 and 65, and (iii) have not been admitted to any clinical setting during the study. Participants with cognitive deficiencies were excluded from the study. We aimed to create a sample that matched the age and sex distribution of the population. To achieve this, six age-sex groups (18–29, 30–44, 45–65 years; men and women) were used (Supplementary Table S1).

A multi-stage sampling method was employed. In the first stage, we selected representative geological units within each region of the country. Mongolia has four geographical regions, each with 5–6 prefectures. Geopolitical units studied were the capital city, Ulaanbaatar, and the following eight prefectures: Gobi-Altai, Khovd (Western region), Uvurkhangai, Arkhangai (Mountain region), Tuv, Dornogobi (Central region), Dornod and Sukhbaatar (Eastern region). In the second stage, the participants were recruited from 48 sampling centers, including 24 centers in 8 districts of Ulaanbaatar and 24 centers in 8 prefectures of the four rural regions. In the final stage, two or three individuals from each age-sex group were randomly selected from each sampling center depending on the population density. If selected participants were unavailable at the center, they were replaced by the following available participants regardless of age and sex category.

**Figure 1:**
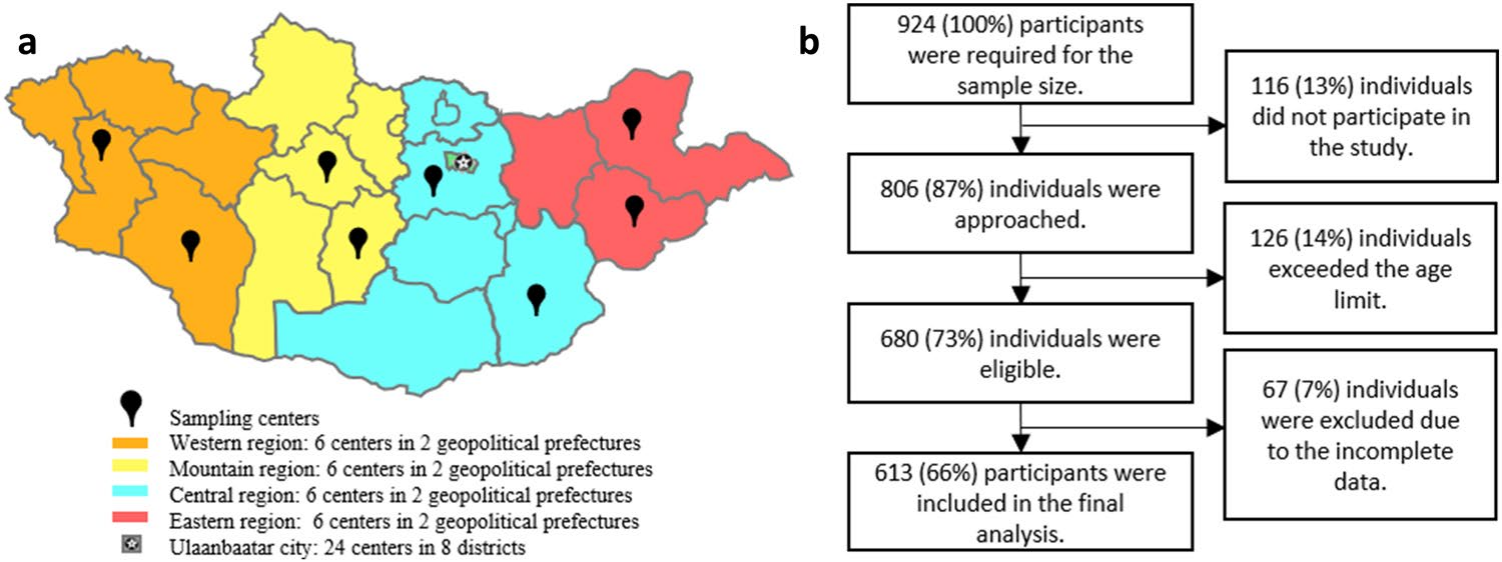
Study flowchart and sampling sites. (**a**) Sampling sites: Out of 48 sampling centers, 24 were in 8 districts of Ulaanbaatar (urban areas) and 24 were in 8 prefectures of 4 rural regions (rural areas). (**b**) Study flowchart.

Figure 1 shows the flowchart of the study.

Of the 924 invited individuals, 118 did not attend the sampling center, leaving 806 participants to be approached for the study. 126 of those approached were ineligible due to exceeding the age limit, and 67 were excluded due to missing data on key demographic variables. The remaining 613 participants were included in the final analysis.

### Data collection

The data collection for this study commenced on September 7, 2020 and concluded on December 22, 2020. The investigation was conducted in Mongolian, the official language of Mongolia. Trained research personnel or medical doctors delivered in-person explanations of the study instruments to the participants, and assisted them in providing responses via tablet devices. Additionally, comprehensive data encompassing participants' demographic characteristics and lifestyle factors were gathered. Moreover, to evaluate the effect of obesity, anthropometric measurements comprising height, weight, waist circumference, and neck circumference were obtained^21^. To assess the prevailing physical health status, four primary vital signs were noninvasively examined by adept research personnel or medical doctors: body temperature measured at the forehead or wrist with an electronic infrared thermometer (Tida, TD-133, China), blood pressure and heart rate assessed using an advanced blood pressure monitor (BP A6 PC, Microlife, Switzerland), and arterial oxygen saturation (SpO2) determined via pulse oximetry (PO40, Beurer, Germany). Blood pressure, heart rate, and arterial oxygen saturation were assessed in accordance with the WHO’s guidelines on the measurement of these vital signs^22,23^.

### Instruments

#### The Hospital Anxiety and Depression Scale (HADS)

The 14-item HADS was used as a reliable and valid measure of anxiety (HADS-A, 7 items) and depression (HADS-D, 7 items). It was developed to identify mental health symptoms in the past week in nonclinical and clinical settings^17^. Each item is evaluated on a 4-point scale, resulting in a total score ranging from 0 to 21 for each subscale. Scores falling within the range of 0–7 are classified as normal, scores between 8 and 10 indicate a mild abnormality, scores ranging from 11 to 14 denote a moderate abnormality, and scores from 15 to 21 represent severe abnormality for each subscale. We determined the psychometric properties of the HADS in the general population in our previous study, which demonstrated that the Mongolian version of HADS has good validity and reliability for assessing anxiety and depression^18^.

#### The Brain Overwork Scale (BOS-10)

The BOS-10 is a 10-item self-report questionnaire that was developed to assess brain overwork in the general population. The BOS-10 measures three dimensions of brain overwork: excessive thinking, hypersensitivity, and restless behavior (Supplementary Table S2). To score the BOS-10, participants are asked to rate how often they have experienced each of the 10 symptoms in the past week on a scale of 0 (never) to 3 (very often). The total score is the sum of the ratings for all 10 items. Higher scores indicate greater levels of brain overwork. The scale has been shown to have good psychometric properties, including high internal consistency, test–retest reliability, and construct validity. The BOS-10 is a tool for screening for brain overwork in the general population and can be used to identify individuals who may need further assessment or treatment^19^.

### Statistical analysis

Data were presented as mean ± standard deviation (SD). Differences between the two groups were examined using the χ^2^ test for categorical data and one-way ANOVA or t-test for continuous data, as appropriate. To estimate the prevalence of anxiety and depression, cut-off points of 8 and 11 were used for the HADS-A and HADS-D, respectively. The cut-off points for each subscale and total score of the BOS-10 were established by dichotomizing the scores at 1 SD above the mean of each score in the participants of this study^24^. Correlation analyses between continuous variables were performed using Pearson’s bivariate test. Multiple linear regression analyses were used to determine if risk factors (independent variables: all variables, including sociodemographic characteristics, body measurements, vital function indexes, and the HADS scores) were associated with the mean scores of the BOS-10 (dependent variables: excessive thinking, hypersensitivity, restless behavior, and the total scores of BOS-10). Multicollinearity was examined using variance inflation factor (VIF) and tolerance (1 < VIF < 2.5; tolerance < 10). Homoscedasticity was assessed using scatter plots of residuals by predicted values. No outliers were detected (Cook’s distance < 1; standard residuals <  ± 3.3). The independence assumption was tested using the Durbin–Watson coefficient (satisfied if 1.5 < Durbin–Watson < 2.5). To construct a receiver operating characteristic (ROC) curve, the dichotomized variable at the HADS total score with a cut-off point of 13 was used for BOS-10, whereas the dichotomized variable at the BOS-10 total score with a cut-off point of 28 was used for HADS. Using the new nominal variables, we evaluated the screening ability of the HADS and BOS-10 at a range of cut-off points (Supplementary Table S3). All statistical tests were two-tailed with a statistical significance set at p < 0.05. Data were analyzed using SPSS v26.0 and JAMOVI v2.2.5.

### Ethical considerations

All procedures performed in this study were done so in accordance with the ethical standards of the institutional and/or national research committee and the 1964 Helsinki Declaration and its later amendments. The design and methods were reviewed and approved by the Institutional Review Board and Ethics Committee at the Mongolian National University of Medical Sciences, Ulaanbaatar, Mongolia (number: 20/03-05). Written informed consent was obtained from all participants.

## Results

There were 613 participants (190 men and 423 women) aged 18–65 years, with a mean ± SD of 41.8 ± 12.4 years. The details of the sociodemographic characteristics are described in Table 1.

**Table 1:** Sociodemographic characteristics of the participants by sex.

Characteristics, n (%)	Total	Male	Female
Total	613 (100)	190 (31)	423 (69)
Age group			
18–29	118 (19.2)	30 (15.8)	88 (20.8)
30–44	244 (39.8)	64 (33.7)	180 (42.6)
45–65	251 (40.9)	96 (50.5)	155 (36.6)
Marital status			
Never-married	90 (14.7)	34 (17.9)	56 (13.2)
Others^#^	71 (11.6)	15 (7.9)	56 (13.2)
Married	452 (73.7)	141 (74.2)	311 (73.5)
Education			
Middle school and below	271 (44.2)	95 (50.0)	176 (41.6)
Associate’s degree	157 (25.6)	46 (24.2)	111 (26.2)
Bachelor’s degree	163 (26.6)	44 (23.2)	119 (28.1)
Master’s degree and above	22 (3.6)	5 (2.6)	17 (4.0)
Employment			
Unemployed	96 (15.7)	20 (10.5)	76 (15.7)
Student	36 (5.9)	20 (10.5)	16 (5.9)
Pensioner	134 (21.9)	43 (22.6)	91 (21.9)
Employed	347 (56.6)	107 (56.3)	240 (56.7)
Income			
< ₮500,000	393 (64.1)	115 (60.5)	278 (65.7)
₮500,001–₮1,000,000	213 (34.7)	73 (38.4)	140 (33.1)
> ₮1,000,000	7 (1.1)	2 (1.1)	5 (1.2)
Living condition			
Ger (traditional pelt tent)	407 (66.4)	125 (65.8)	282 (66.7)
Apartment	206 (33.6)	65 (34.2)	141 (33.3)
Residency location			
Rural areas	341 (55.6)	116 (61.1)	225 (53.2)
Urban areas	272 (44.4)	74 (38.9)	198 (46.8)
Alcohol use			
Yes	171 (29.3)	50 (27.8)	122 (30.0)
No	415 (70.7)	130 (72.2)	285 (70.0)
Tobacco use			
Yes	123 (20.6)	48 (25.9)	75 (18.2)
No	464 (77.6)	132 (71.4)	332 (80.4)
Had smoked before	11 (1.8)	5 (2.7)	6 (1.5)
Continuous variables, mean ± SD			
Age	41.8 ± 12.4	43.4 ± 12.6	41.1 ± 12.3
Vital function indices			
Body temperature	36.5 ± 0.3	36.5 ± 0.3	36.4 ± 0.3
Heart rate	78.2 ± 11.3	77.9 ± 12.9	78.3 ± 10.6
Arterial systolic pressure	126.0 ± 21.6	131.0 ± 20.0	123.0 ± 21.9
Arterial diastolic pressure	80.2 ± 13.6	83.7 ± 13.6	78.6 ± 13.3
Arterial oxygen saturation	95.2 ± 2.0	95.3 ± 2.0	95.1 ± 2.0
Body measurements			
Body mass index (BMI)	27.1 ± 5.3	27.3 ± 5.4	27.0 ± 5.2
Neck circumference	34.6 ± 3.5	35.5 ± 3.8	34.1 ± 3.2
Waist circumference	89.4 ± 13.8	91.1 ± 13.2	88.7 ± 14.0

^#^Others included remarried, co-habiting, separated, divorced, and widowed. ₮: Mongolian tugrik (MNT₮), US $1 = MNT ₮2850. n: number. SD: standard deviation. Missing values: alcohol use (26), tobacco use (15), heart rate (9), arterial systolic pressure (10), arterial diastolic pressure (16), arterial oxygen saturation (27), BMI (5), neck circumference (7), waist circumference (9).

There were no differences in marital status, education, income, living condition, residency location, alcohol use, body temperature, heart rate, oxygen saturation, and body mass index (BMI) between men and women. Women were younger, more unemployed, smoked less, and had less blood pressure and neck circumference. Of the participants, 64.1% earned less than ₮500,000 (low income), 34.7% received ₮500,000–1,000,000 (middle income), and 1.1% received more than ₮1,000,000 (high income). 33.6% of the sample population lived in traditional gers or houses that were not connected with water, sanitation, and heating system. More than half (55.6%) of the sample population lived in rural areas.

Table 2 shows the mean and SD of each domain of the HADS and BOS-10 by sociodemographic characteristics among the participants.

**Table 2:** Normative values of the HADS and BOS-10 scores by sociodemographic characteristics.

Characteristics (n)	HADS (mean ± SD)			BOS-10 (mean ± SD)			
	A	D	T	ET	H	RB	T
Total, unadjusted (613)	6.0 ± 3.0	5.6 ± 2.7	11.6 ± 4.9	7.0 ± 2.2	5.4 ± 2.4	8.3 ± 3.9	20.7 ± 7.3
Total, age and sex adjusted (613)	9.2 ± 4.2	8.2 ± 3.7	17.3 ± 6.6	10.7 ± 3.1	8.4 ± 3.0	12.6 ± 4.8	31.0 ± 9.4
Male (190)							
18–29 (30)	8.2 ± 3.4	7.0 ± 3.0	15.1 ± 5.3	8.0 ± 2.7	7.6 ± 2.1	10.3 ± 3.6	25.9 ± 7.9
30–44 (64)	6.1 ± 3.0	5.4 ± 2.2	11.5 ± 4.3	7.3 ± 2.0	5.4 ± 2.3	9.2 ± 3.4	21.9 ± 6.2
45–65 (96)	5.4 ± 2.6	5.3 ± 2.4	10.7 ± 4.4	6.4 ± 2.0	4.7 ± 1.9	7.0 ± 3.9	18.0 ± 6.6
Total (190)	6.0 ± 3.0	5.6 ± 2.5	11.6 ± 4.8	7.0 ± 2.2	5.4 ± 2.5	8.2 ± 3.9	20.6 ± 7.3
All male, age-adjusted (190)	13.6 ± 6.1	12.0 ± 5.1	25.5 ± 9.3	14.5 ± 4.6	12.4 ± 4.1	18.3 ± 6.9	45.0 ± 13.8
Female (453)							
18–29 (88)	6.3 ± 3.4	5.5 ± 3.1	11.8 ± 5.5	7.8 ± 2.3	6.0 ± 2.5	10.6 ± 3.5	24.4 ± 6.7
30–44 (180)	6.4 ± 3.3	6.0 ± 2.8	12.3 ± 5.3	7.4 ± 2.0	6.2 ± 2.5	9.5 ± 3.8	23.0 ± 7.0
45–65 (155)	5.5 ± 2.4	5.3 ± 2.5	10.8 ± 3.9	6.0 ± 1.8	4.1 ± 1.9	5.9 ± 2.8	16.0 ± 5.4
Total (423)	6.0 ± 3.1	5.6 ± 2.8	11.6 ± 5.0	7.0 ± 2.2	5.4 ± 2.4	8.4 ± 3.9	20.8 ± 7.4
All female, age-adjusted (423)	4.8 ± 2.4	4.4 ± 2.2	9.2 ± 3.9	5.6 ± 1.6	4.3 ± 1.8	7.0 ± 2.7	17.0 ± 5.0
Marital status							
Never-married (90)	6.8 ± 3.8	5.6 ± 2.9	12.4 ± 5.9	7.9 ± 2.3	6.3 ± 2.8	9.8 ± 3.7	24.0 ± 7.3
Others* (71)	5.9 ± 3.2	5.3 ± 2.9	11.2 ± 5.2	7.0 ± 10.8	5.0 ± 2.5	7.6 ± 4.3	19.6 ± 7.9
Married (452)	5.9 ± 2.8	5.7 ± 2.6	11.6 ± 4.6	6.8 ± 12.8	5.3 ± 2.3	8.2 ± 3.8	20.2 ± 7.1
Education							
Middle school and below (271)	5.9 ± 2.9	5.5 ± 2.7	11.5 ± 4.8	6.6 ± 2.2	5.1 ± 2.3	7.7 ± 3.9	19.4 ± 7.2
Associate’s degree (157)	5.8 ± 2.8	5.5 ± 2.4	11.3 ± 4.5	6.9 ± 2.1	5.3 ± 2.3	8.0 ± 4.0	20.1 ± 7.3
Bachelor’s degree (163)	6.4 ± 3.5	5.9 ± 2.9	12.3 ± 5.5	7.6 ± 2.0	6.1 ± 2.5	9.6 ± 3.6	23.2 ± 7.1
Master’s degree and above (22)	5.6 ± 2.8	5.3 ± 2.6	11.0 ± 4.9	7.1 ± 2.2	5.5 ± 2.3	9.5 ± 3.0	22.1 ± 6.0
Employment							
Unemployed (96)	5.8 ± 2.9	5.5 ± 2.8	11.3 ± 5.0	7.1 ± 2.2	5.2 ± 2.2	8.5 ± 4.4	20.8 ± 7.8
Student (36)	7.0 ± 3.5	5.7 ± 2.8	12.7 ± 5.0	8.1 ± 2.4	7.1 ± 3.3	10.9 ± 3.9	26.1 ± 7.5
Pensioner (134)	5.5 ± 2.7	5.3 ± 2.7	10.8 ± 4.7	6.0 ± 2.0	4.3 ± 1.9	6.0 ± 3.1	16.3 ± 5.9
Employed (347)	6.2 ± 3.1	5.8 ± 2.6	11.9 ± 4.9	7.2 ± 2.1	5.7 ± 2.4	8.9 ± 3.7	21.8 ± 6.9
Income							
< ₮500,000 (393)	6.0 ± 3.0	5.4 ± 2.7	11.4 ± 4.8	6.8 ± 2.2	5.1 ± 2.4	7.9 ± 4.0	19.8 ± 7.4
₮500,001–₮1,000,000 (213)	6.1 ± 3.1	6.0 ± 2.7	12.1 ± 5.0	7.3 ± 2.1	5.9 ± 2.4	9.2 ± 3.7	22.4 ± 7.0
> ₮1,000,001 (7)	6.1 ± 4.9	4.4 ± 3.1	10.6 ± 6.6	6.4 ± 1.9	4.3 ± 2.0	7.4 ± 3.1	18.1 ± 5.9
Living condition							
Ger (407)	5.9 ± 2.8	5.4 ± 2.6	11.3 ± 4.6	6.7 ± 2.1	5.1 ± 2.3	7.6 ± 3.9	19.3 ± 7.3
Apartment (206)	6.3 ± 3.5	6.0 ± 2.8	12.4 ± 5.4	7.5 ± 2.1	6.0 ± 2.4	9.9 ± 3.5	23.5 ± 6.6
Residency location							
Rural areas (341)	5.8 ± 2.7	5.4 ± 2.6	11.2 ± 4.4	6.4 ± 2.2	4.9 ± 2.4	7.1 ± 3.8	18.3 ± 7.3
Urban areas (272)	6.3 ± 3.4	5.9 ± 2.8	12.2 ± 5.4	7.7 ± 1.9	6.0 ± 2.3	10.0 ± 3.3	23.7 ± 6.1
Alcohol use							
Yes (172)	5.8 ± 3.0	5.7 ± 2.8	11.5 ± 5.0	7.4 ± 2.3	5.6 ± 2.4	9.2 ± 3.8	22.1 ± 7.1
No (415)	6.1 ± 3.0	5.6 ± 2.6	11.7 ± 4.8	6.8 ± 2.1	5.3 ± 2.4	8.0 ± 3.9	20.1 ± 7.3
Tobacco use							
Yes (123)	6.0 ± 3.2	5.5 ± 2.7	11.4 ± 5.0	7.1 ± 2.3	5.1 ± 2.1	8.6 ± 3.8	20.8 ± 7.0
No (464)	6.0 ± 3.0	5.7 ± 2.7	11.7 ± 4.9	6.9 ± 2.2	5.4 ± 2.5	8.3 ± 3.9	20.6 ± 7.4
Had smoked before (11)	7.5 ± 2.8	6.6 ± 2.3	14.0 ± 4.6	8.4 ± 1.8	7.2 ± 2.0	10.8 ± 3.9	26.4 ± 7.7
BMI ranges							
Normal (233)	6.2 ± 3.3	5.8 ± 2.6	11.9 ± 5.1	7.4 ± 2.2	5.9 ± 2.4	9.4 ± 3.9	22.7 ± 7.2
Overweight (196)	5.8 ± 2.6	5.6 ± 2.9	11.6 ± 4.7	6.8 ± 2.2	5.2 ± 2.4	8.2 ± 3.9	20.3 ± 7.3
Obesity (170)	6.0 ± 2.9	5.6 ± 2.7	11.5 ± 4.7	6.4 ± 2.0	4.9 ± 2.3	7.0 ± 3.6	18.3 ± 6.7

^#^Others included remarried, co-habiting, separated, divorced, and widowed. A: anxiety score, HADS. *BMI* body mass index. *BOS-10* Brain Overwork Scale-10. D: depression score, HADS. *ET* excessive thinking score, BOS-10. H: hypersensitivity score, BOS-10. *HADS* Hospital Anxiety and Depression Scale. *n* count. *RB* restless behavior score, BOS-10. *SD* standard deviation. *T* total score.

The age- and sex-adjusted mean scores of HADS were 9.2 for anxiety, 8.2 for depression, and 17.3 for the total score. The age- and sex-adjusted mean scores of BOS were 10.7 for excessive thinking, 8.4 for hypersensitivity, 12.6 for restless behavior, and 31.0 for the total score. There were no differences in the HADS scores between sex, education, employment, income, alcohol use, tobacco use, and BMI ranges among the participants. In contrast, there were significant differences in the BOS-10 scores between all variables, except sex (p < 0.001, one-way ANOVA or *t*-test). Both in HADS and BOS-10, younger participants had higher scores than older participants and participants living in urban areas had higher scores than those living in rural areas (both p < 0.001, *t*-test). Participants living in apartments had higher scores than the other participants in the HADS depression (p = 0.007, *t*-test). Furthermore, subgroup analyses revealed that as age decreased, all the HADS and BOS-10 scores dropped among the male participants (all p < 0.05 or p < 0.001, one-way ANOVA), and all scores excluding depression decreased among female participants (p = 0.060, one-way ANOVA).

The prevalence of anxiety, depression, and brain overwork were described as percentages of participants above the cut-off scores of the HADS and BOS-10 (Table 3).

**Table 3:** Prevalence of anxiety, depression, and brain overwork by sex and age group, n = 613.

Characteristics (n)	HADS (cut-off score)						HADS (cut-off score)						BOS-10 (cut-off score)							
	A (8)		D (8)		T (17)		A (11)		D (11)		T (23)		ET (9)		H (8)		RB (12)		T (28)	
	n	%	n	%	n	%	n	%	n	%	n	%	n	%	n	%	n	%	n	%
Male(190)																				
18–29 (30)	19	63.3	13	43.3	16	53.3	7	23.3	4	13.3	1	3.3	10	33.3	12	40.0	9	30.0	11	36.7
30–44 (64)	21	32.8	10	15.6	8	12.5	4	6.3	1	1.6	0	0	10	15.6	7	10.9	12	18.8	8	12.5
45–65 (96)	21	21.9	4	14.6	8	8.3	3	3.1	1	1.0	0	0	7	7.3	4	4.2	12	12.5	10	10.4
Total (190)	61	32.1	37	19.5	32	16.8	14	7.4	6	3.2	1	0.5	27	14.2	23	12.1	33	17.4	29	15.3
Age-adjusted %	38.8		20.3		23.6		10.5		5.0		1.0		18.5		17.7		20.3		19.2	
p value	< 0.001		0.002		< 0.001		< 0.001		0.002		0.069		0.002		< 0.001		0.082		0.002	
Female(423)																				
18–29 (88)	33	37.5	24	27.3	21	23.9	12	13.6	4	4.5	2	2.3	21	23.9	13	14.8	30	34.1	25	28.4
30–44 (180)	63	35	51	28.3	38	21.1	22	12.2	10	5.6	7	3.9	28	15.6	27	15.0	41	22.8	41	22.8
45–65 (155)	30	19.4	27	17.4	15	9.7	4	2.6	7	4.5	0	0	7	4.5	6	3.9	6	3.9	3	1.9
Total (423)	126	29.8	102	24.1	74	17.5	38	9.0	21	5.0	9	2.1	56	13.2	46	10.9	77	18.2	69	16.3
Age-adjusted %	30.5		19.7		18.1		9.4		4.9		2.1		14.2		11.2		19.7		17.4	
p value	0.002		0.049		0.005		0.002		0.890		0.048		< 0.001		0.002		< 0.001		< 0.001	
Total (613)																				
18–29 (118)	52	44.1	37	31.4	37	31.4	19	16.1	8	6.8	3	2.5	31	26.3	25	21.2	39	33.1	36	30.5
30–44 (244)	84	34.4	61	25.0	46	18.9	26	10.7	11	4.5	7	2.9	38	15.6	34	13.9	53	21.7	49	20.1
45–65 (251)	51	20.3	41	16.3	23	9.2	7	2.8	8	3.2	0	0	14	5.6	10	4.0	18	7.2	13	5.2
Total (613)	187	30.5	139	22.7	106	17.3	52	8.5	27	4.4	10	1.6	83	13.5	69	11.3	110	17.9	98	16.0
Age-adjusted %	34.5		20.0		20.8		9.9		4.9		1.6		16.3		14.4		20.0		18.3	
p value	< 0.001		0.003		< 0.001		< 0.001		0.291		0.029		< 0.001		< 0.001		< 0.001		< 0.001	

*p values were analyzed using the *χ*^2^ test. A: anxiety score, HADS. BOS-10: Brain Overwork Scale-10. D: depression score, HADS. ET: excessive thinking score, BOS-10. H: hypersensitivity score, BOS-10. HADS: Hospital Anxiety and Depression Scale. n: count. RB: restless behavior score, BOS-10. T: total score. The cut-off scores of the HADS were 8 (including mild, moderate, and severe levels) and 11 (including moderate and severe levels) for both anxiety and depression. The cut-off scores of the BOS-10 using the 1 standard deviation above the mean criterion were 9 for ET, 8 for H, 12 for RB, and 28 for the total score.

The cut-off scores of the HADS were 8 (including mild, moderate, and severe levels) and 11 (including moderate and severe levels) for both anxiety and depression. The cut-off scores of the BOS-10, using a criterion of 1 standard deviation above the mean, were 9 for ET, 8 for H, 12 for RB, and 28 for the total score. The prevalence was 9.9–34.5% for anxiety, 4.9–20% for depression, and 18.3% for brain overwork (the total score of the BOS-10). There was no difference in the prevalence of the HADS and BOS-10 scores between the sexes. In contrast, the prevalence of the HADS and BOS-10 scores differed between age groups. Younger participants had higher anxiety, depression, and brain overwork than older participants.

Table 4 shows the Pearson’s correlation coefficients between the HADS and BOS-10 scores and selected continuous variables.

**Table 4:** Pearson correlation coefficients of the HADS and BOS-10 scores with risk factors, n = 613.

Variables	HADS			BOS-10			
	A	D	T	ET	H	RB	Total
Age	− 0.174***	− 0.097*	− 0.161*	− 0.365***	− 0.391***	− 0.457***	− 0.481***
Body temperature	0.026	− 0.002	0.015	− 0.088*	− 0.029	− 0.063	− 0.069
Heart rate	0.109*	0.043	0.091*	0.076	0.073	0.071	0.085*
Arterial systolic pressure	0	− 0.092*	− 0.05	− 0.134***	− 0.145***	− 0.174***	− 0.18***
Arterial diastolic pressure	− 0.003	− 0.118	− 0.066	− 0.119**	− 0.114**	− 0.167***	− 0.162***
Arterial oxygen saturation	0.028	0.013	0.025	0.09*	0.04	0.05	0.066
BMI	− 0.011	0	− 0.007	− 0.179***	− 0.186***	− 0.22***	− 0.232***
Neck circumference	− 0.021	− 0.045	− 0.037	− 0.075	− 0.133**	− 0.1*	− 0.119**
Waist circumference	− 0.033	− 0.029	− 0.036	− 0.201***	− 0.239***	− 0.27***	− 0.283***
HADS-A				0.420***	0.383***	0.262***	0.391***
HADS-D				0.278***	0.317***	0.194***	0.291***
HADS-T				0.413***	0.412***	0.269***	0.402***

*p < 0.05, **p < 0.01, ***p < 0.001. p values were calculated using Pearson’s bivariate correlation. A: anxiety score, HADS. BOS-10: Brain Overwork Scale-10. D: depression score, HADS. ET: excessive thinking score, BOS-10. H: hypersensitivity score, BOS-10. HADS: Hospital Anxiety and Depression Scale. RB: restless behavior score, BOS-10. T: total score.

The HADS and BOS-10 scores showed significant positive correlations to each other and the coefficients ranged from 0.194 to to 0.420. Age was inversely correlated with both HADS and BOS-10 scores. Anxiety was correlated with heart rate. In contrast, depression was inversely correlated with arterial systolic pressure. The BOS-10 scores were inversely correlated to all risk factors, with the exception of heart rate.

Table 5 shows the coefficients of multiple linear regression for the BOS-10.

**Table 5:** Multiple linear regression analyses on the BOS-10 scores by risk factors, n = 613.

Variables	B	Beta	t	p value*	95% Confidence Interval for Exp (B)		Collinearity Statistics	
					Lower	Upper	Tolerance	Variance Inflation Factor
Excessive thinking, BOS-10 (R^2^ = 0.339, D-W = 1.92, Cook’s = 0.003, B-P = 0.183, F_27_ = 9.63, p < 0.001)								
Constant	20.42	10.70	1.87	0.062	− 1.03	41.03		
Age	− 0.02	0.01	− 2.64	0.009	− 0.04	− 0.01	0.66	1.66
Residency	− 0.75	0.19	− 3.93	< 0.001	− 1.13	− 0.38	0.82	1.22
Alcohol use	0.44	0.18	2.48	0.013	0.09	0.79	0.96	1.04
Anxiety score	0.23	0.03	7.72	< 0.001	0.17	0.29	0.87	1.15
Depression score	0.08	0.03	2.37	0.018	0.01	0.14	0.87	1.15
Hypersensitivity, BOS-10 (R^2^ = 0.324, D-W = 2.02, Cook’s = 0.004, B-P = 0.032, F_27_ = 10.3, p < 0.001)								
Constant	16.58	11.98	1.38	0.167	− 6.95	40.11		
Age	− 0.05	0.01	− 4.61	< 0.001	− 0.07	− 0.03	0.66	1.51
Residency	− 0.49	0.21	− 2.29	0.023	− 0.91	− 0.07	0.82	1.22
Anxiety score	0.19	0.03	5.55	< 0.001	0.12	0.25	0.87	1.15
Depression score	0.15	0.04	3.90	< 0.001	0.07	0.22	0.87	1.15
Restless behavior, BOS-10 (R^2^ = 0.355, D-W = 2.04, Cook’s = 0.002, B-P = 0.075, F_27_ = 13.0, p < 0.001)								
Constant	30.90	19.21	1.6	0.108	− 6.85	68.67		
Age	− 0.08	0.02	− 4.77	< 0.001	− 0.11	− 0.05	0.66	1.51
Residency	− 1.90	0.34	5.52	< 0.001	− 2.57	− 1.22	0.82	1.22
Alcohol use	0.86	0.32	2.70	0.007	1.24	1.49	0.96	1.04
Anxiety score	0.20	0.05	3.78	< 0.001	0.10	0.31	0.87	1.15
Total score, BOS-10 (R^2^ = 0.429, D-W = 2.08, Cook’s = 0.003, B-P = 0.024, F_27_ = 14.1, p < 0.001)								
Constant	67.33	33.64	2.03	0.046	2.26	134.40		
Age	− 0.15	0.03	− 5.20	< 0.001	− 0.22	− 0.10	0.66	1.51
Employment	3.23	1.50	2.16	0.031	0.29	6.17	0.80	1.24
Income	4.39	2.24	1.97	0.049	− 0.01	8.79	0.87	1.14
Residency location	− 3.15	0.60	− 5.22	< 0.001	− 4.33	− 1.96	0.82	1.22
Alcohol use	1.29	0.56	2.30	0.022	0.19	2.39	0.96	1.04
Anxiety score	0.62	0.09	6.59	< 0.001	0.43	0.81	0.87	1.15
Depression score	0.29	0.10	2.84	0.005	0.09	0.51	0.87	1.15

*p values were tested using the multiple linear regression. Predictors: (constant), sex, age, marital status, education, employment, income, living condition (ger–apartment), residency (rural–urban), alcohol use, tobacco use, body temperature, heart rate, systolic blood pressure, diastolic blood pressure, arterial oxygen saturation, body mass index, neck circumference, waist circumference, anxiety score, depression score. *B-P* Breusch-Pagan, *D-W* Durbin-Watson.

To investigate how brain overwork was associated with the sociodemographic characteristics, independent variables were selected for each subscale and the total score of the BOS-10, using a stepwise method. This method demonstrates that brain overwork (the total score of the BOS-10) was associated by decreased age, unemployment (student), less income, residency location (apartment), alcohol use, anxiety, and depression (R^2^ = 0.429, p < 0.001). No multicollinearity was detected between the tested variables; the independence assumption was satisfied; the distribution of the residuals satisfied the normality assumptions. The variance of the model was constant, and homoscedasticity was not violated.

**Figure 2:**
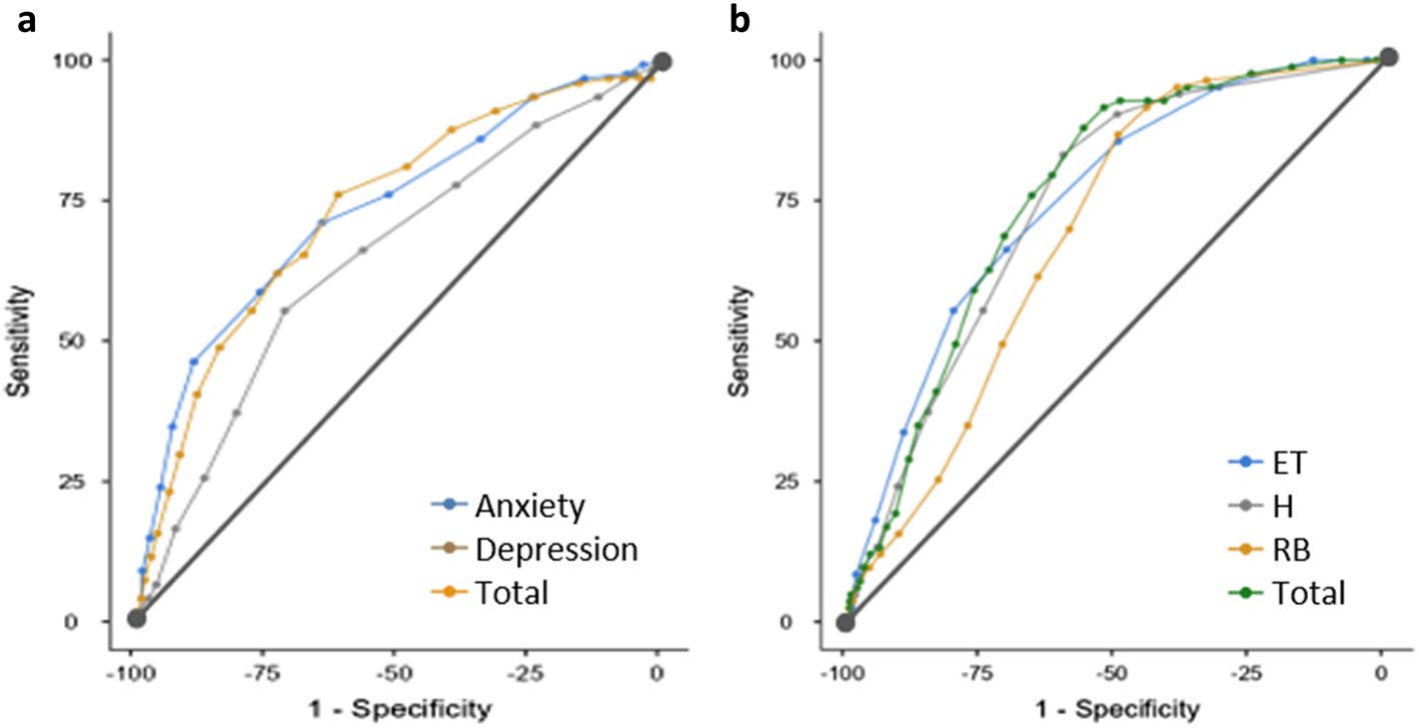
ROC curves of the HADS and BOS-10. (**a**) The true-positive rates of the HADS subscales against the false-positive rates were plotted at a variety of thresholds for a single variable created by dichotomizing the BOS-10 total score with the cut-off score of 28. (**b**) The true-positive rates of the BOS-10 scores against the false-positive rates were plotted at a variety of thresholds for a single variable created by dichotomizing the HADS total score with the cut-off score of 17.

Figure 2 shows the ROC curves of the HADS and BOS-10.

The area under the ROC curve (AUC) values of the HADS were 0.73, 0.65, and 0.73 for anxiety, depression, and the total score, respectively. AUC values of the BOS-10 were 0.75, 0.74, 0.69, and 0.76 for ET, H, RB, and the total score, respectively. The AUC indicates performance; the greater the AUC, the better the performance. Two cut-off points were set to calculated sensitivity, specificity, positive predictive value, negative predictive value, and Youden’s index for the HADS and BOS-10 (Supplementary Table S3).

## Discussion

The present study aimed to investigate the prevalence, risk factors, and interrelationships of anxiety, depression, and brain overwork among the Mongolian adult population. This is the first report on the normative data on anxiety, depression, and brain overwork in Mongolia, shedding light on the sociodemographic characteristics associated with these mental health conditions and providing insights into potential risk factors. The study sample was chosen to represent the target population which is composed of urban and rural residents. To measure anxiety and depression, we employed the HADS, whereas to assess brain overwork, we used the BOS-10 for excessive thinking, hypersensitivity, and restless behavior. In our previous studies, we validated the Mongolian versions of both the HADS and BOS-10, which showed good validity and reliability for assessing brain overwork in the general population. Therefore, the current results provide valuable information on mental health in the general population of Mongolia.

The prevalence of anxiety, depression, and brain overwork in the study population was significant, with prevalence rates ranging from 9.9 to 34.5% for anxiety, 4.9 to 20% for depression, and 18.3% for brain overwork. The normative scores were higher or similar compared with those of other countries^25–30^ (Supplementary Table S4). These findings highlight the substantial burden of these mental health conditions within the population and emphasize the need for targeted interventions and support services.

The study also explored the relationship between sociodemographic characteristics and mental health outcomes. The results indicated that there were no significant differences in marital status, education, income, living condition, residency location, alcohol use, body temperature, heart rate, oxygen saturation, and BMI between men and women. However, notable distinctions were observed in age, employment status, smoking habits, blood pressure, and neck circumference. These differences suggest that certain factors, such as age and employment, may contribute to variations in mental health outcomes. While no significant differences were observed between the sexes in terms of prevalence rates, variations were evident across different age groups. Younger participants consistently displayed higher levels of anxiety, depression, and brain overwork compared to older individuals. This finding suggests that specific age-related factors, such as life stressors, social pressures, and changes in social roles, may contribute to higher brain overwork in younger age groups. Younger individuals, particularly women, exhibited higher levels of anxiety, depression, and brain overwork, which may be attributed to the challenges associated with transitions and societal expectations. Additionally, the inverse correlations between age and both HADS and BOS-10 scores highlight the potential protective effect of age against the development of anxiety, depression, and brain overwork. This finding may be attributed to increased coping skills, resilience, and adaptive strategies developed over the lifespan.

We found that residency location plays an important role in mental health conditions. The urban residents showed higher scores in anxiety, depression, and brain overwork compared to the rural residents. Initially, we anticipated that individuals residing in rural areas would experience higher levels of anxiety and depression compared to those living in urban areas due to the harsh living conditions, as suggested by previous studies^31–33^. However, our findings were contrary to our initial expectations. The environmental difference might be related to the climate and living conditions. The climate in Mongolia is harsh, with long, cold winters and short, hot summers. The average temperature in January is -30 °C, and the average temperature in July is 20 °C. Over half of the population, approximately 3 million people, resides in rural areas, engaging in nomadic herding and living in traditional dwellings known as gers. Gers lack essential amenities such as centralized electricity, water, sanitation, and heating systems. In contrast, urban residents, mainly concentrated in the capital city of Ulaanbaatar, reside in apartments that are connected to centralized utilities, including heating systems for the harsh winter months. Since the democratic revolution in 1990, the rural population has migrated to Ulaanbaatar, resulting in a population surpassing one million residents. Consequently, this migration has led to the expansion of ger areas in Ulaanbaatar, contributing to its notoriety as one of the most air-polluted cities in the world^34^. While some households within Ulaanbaatar's ger areas have transitioned to conventional houses, comprehensive central utility systems, apart from electricity, remain lacking. Our previous study found that urban dwellers, particularly those in ger areas, experience lower physical quality of life compared to their rural counterparts^35^. These findings emphasize the need for targeted interventions and support services for urban dwellers, particularly who live in ger areas.

The study also revealed significant correlations between the HADS and BOS-10 scores. The positive correlations observed between the two scales suggest that individuals experiencing higher levels of anxiety and depression may also exhibit brain overwork and vice versa. This finding suggests potential shared underlying mechanisms and supports the notion of a transdiagnostic approach to mental health assessment and treatment. Also, anxiety was found to be positively correlated with heart rate, while depression demonstrated an inverse correlation with arterial systolic pressure. However, these findings are not generalizable and emphasize the need for further research on the complex relationship between mental and physical health in diverse populations and longitudinal designs.

Moreover, the study identified several sociodemographic factors associated with an increased risk of brain overwork, reflecting the multifaceted nature of this construct. Young age has emerged as a significant risk factor, possibly due to emerging technology-related health concerns. Unemployment/student status and lower income were found to increase risk, suggesting the potential impact of and financial strain on brain function and mental well-being in young adulthood. This aligns with research on the impact of chronic stress and social determinants of health on mental health^9,10^. Interestingly, residency in apartments was also associated with a higher risk, which may be due to factors such as environmental pollution, limited green space, or lack of privacy. In addition, alcohol use was found to be a risk factor of brain overwork, highlighting the complex relationship between substance use and mental health. This result is consistent with our previous findings that an increased risk of alcohol dependence was associated with being unemployed, and having lower levels of education^36^. Notably, both anxiety and depression were also associated with brain overwork, raising questions about the complex interplay between these conditions. While the cross-sectional study cannot establish causation, these findings suggest a need for targeted interventions and support systems for vulnerable groups, such as young people, unemployed individuals, and those facing financial hardship. Future research with longitudinal designs should further explore the complex interplay between sociodemographic factors, emotional dysregulation, and brain overwork to inform more effective prevention and intervention strategies.

The cut-off scores established for the HADS and BOS-10 provide valuable clinical thresholds for identifying individuals with clinically significant levels of anxiety, depression, and brain overwork. These cut-off scores can serve as practical screening tools in various healthcare settings, facilitating early detection and intervention.

Mongolia's mental health service system provides a three-level service. Primary care includes 334 general practice clinics distributed throughout the country, which provide basic mental health services for common disorders and act as gatekeepers. Severe cases are referred to secondary healthcare, which is comprised of general hospitals. For complex diagnoses and non-conventional treatments, patients are further directed to tertiary healthcare. The National Center of Mental Health (NCMH), established in 1961, has 450 beds dedicated to inpatient care and provides a wide range of mental health services, including prevention, early detection, diagnosis, and treatment for the entire population. This structure ensures initial support for common disorders, specialized interventions for complex cases, and dedicated inpatient care at the NCMH. However, the system faces potential resource limitations in budget and service delivery capacity, as there are only 0.6 psychiatrists and 0.5 nurses per 10,000 population, which is lower than the global average^37,38^. Our research provides valuable insights into the prevalence and risk factors of mental health problems, including anxiety, depression, and brain overwork, in Mongolia. Given the high prevalence of mental health problems, the resource limitations of the Mongolian system, and the specific needs identified by our study, it is crucial to advocate for increased funding for mental health services. Such investments should prioritize interventions tailored to the Mongolian context, addressing both psychological and physical aspects of mental well-being. Further research is also warranted to explore the longitudinal trajectories of these mental health conditions and their impact on overall well-being.

Limitations of the study include the reliance on self-report measures, which may be subject to recall bias and social desirability bias. Furthermore, the study was cross-sectional, which means that it cannot establish causality. Additionally, the study had a limited sample size, with a disproportionate number of younger adults and a high attrition rate. Future research should aim to include a broader age range and utilize more objective measures to complement self-report data. Although the findings suggest that the prevalence of anxiety and depression may be high in other developing countries that are undergoing rapid social and economic change such as Mongolia, the study may not be generalizable to other populations.

In conclusion, this study provides valuable insights into the prevalence, risk factors, and interrelationships of anxiety, depression, and brain overwork in a diverse population. The findings underscore the importance of considering sociodemographic factors, including age and employment status, in mental health research and interventions. The observed associations between psychological symptoms and physiological measures highlight the complex interactions between mental and physical health. The identified risk factors and cut-off scores can inform clinical practice and support the development of targeted interventions. Overall, the findings suggest the need for increased funding for mental health services in Mongolia, and interventions that target cognitive processes and emotional reactivity may be effective in reducing mental distress. Further research is warranted to explore the longitudinal trajectories of these mental health conditions and their impact on overall well-being.

## Conclusion

This is the first report on normative data on anxiety, depression, and brain overwork in the general population of Mongolia. The findings of this study suggest that these conditions are a significant problem in the general population of Mongolia. Further research is needed to develop effective interventions to reduce the prevalence and risk factors of anxiety, depression, and brain overwork.

### Supplementary Information

Supplementary Tables.

## Data Availability

The datasets used and/or analysed during the current study available from the corresponding author on reasonable request.
