# Supplementary material for: Anxiety, depression, and brain overwork in the general population of Mongolia
Source: Sci Rep. 2024 Jan 30;14:2484. doi: 10.1038/s41598-024-52779-w (PMC10828419; doi:10.1038/s41598-024-52779-w)
Supplement: Supplementary file 1 — Supplementary Tables. [file 41598_2024_52779_MOESM1_ESM.pdf]

Anxiety, Depression, and Mental Distress in the General Population of Mongolia

Battuvshin Lkhagvasuren[1,2*#], Tetsuya Hiramoto[3#], Enkhjin Bat-Erdene[1,4], Enkhnaran Tumurbaatar[1], Gantsetseg Tumur-Ochir[5], Tsolmontuya Amartuvshin[6], Myagmartseren Dashtseren[6], Edward Lai[7], Vijay Viswanath[8], Takakazu Oka[2], Tsolmon Jadamba[1*]

[1] Brain and Mind Research Institute, Mongolian Academy of Sciences, Ulaanbaatar, Mongolia, [2] Department of Psychosomatic Medicine, International University of Health and Welfare Narita Hospital, Narita, Japan, [3] Department of Psychosomatic Medicine, Fukuoka National Hospital, National Hospital Organization, Fukuoka, Japan, [4] Child Health Institute of New Jersey, Department of Neuroscience and Cell Biology, Robert Wood Johnson Medical School, Rutgers University, New Brunswick, USA, [5] Department of Mental Health Surveillance, National Center for Mental Health, Ulaanbaatar, Mongolia, [6] Department of Family Medicine, School of Medicine, Mongolian National University of Medical Sciences, Ulaanbaatar, Mongolia, [7] School of Osteopathic Medicine, Rowan-Virtua University, Stratford, USA, [8] College of Medicine, University of Cincinnati, Cincinnati, USA

* Corresponding authors: battuvshin@neuroscience.mn (Battuvshin Lkhagvasuren); tsolmon@mas.ac.mn (Tsolmon Jadamba)

Table S1. The number and percentage of the sample in each age-sex category compared with results from the 2020 Population and Housing By-Census of Mongolia.

Age-sex category		Sample size, n (%)		Population, n (%)	
Total		613	(100)	1910630	(100)
	18-29	118	(19.2)	610322	(31.9)
Both sex	30-44	244	(39.8)	780603	(40.9)
	45-65	251	(40.9)	519705	(27.2)
	18-29	30	(4.9)	273476	(14.3)
Male	30-44	64	(10.4)	364514	(19.1)
	45-65	96	(15.7)	282314	(14.8)
	18-29	88	(14.4)	270846	(14.2)
Female	30-44	180	(29.4)	385836	(20.2)
	45-65	155	(25.3)	333644	(17.4)
Residence location	Rural areas	341	(55.6)	860488	(45.0)
	Urban areas	272	(44.4)	1050142	(55.0)

n: number.

Table S2. Brain Overwork Scale – 10 (BOS-10)

The following questions ask about your thoughts and behaviors in your daily life. There are no right or wrong answers, so please answer honestly and to the best of your ability. If you are unsure about how to answer a question, please choose the answer that feels most like you.

For each question, please indicate how often you have felt or behaved this way in the past week.

#	Questions	1	2	3	4	5
Q1	I did not like to have leisure time					
Q2	I tended to overthink even the (most) minor of events					
Q3	I felt tired when reading newspapers / magazines					
Q4	I tended to walk or move fast					
Q5	I tended to stick to one way of doing or thinking about something					
Q6	I felt that others were watching me					
Q7	I was not good at waiting					
Q8	I had racing thoughts (was thinking something a lot)					
Q9	I easily felt tense in public					
Q10	My notebook was full of schedules					
	**Answers**					
1	Very inaccurate / Never (0 day per week)					
2	Moderately inaccurate / Rarely (1-2 day(s) per week)					
3	Neutral / Sometimes (3-4 days per week)					
4	Moderately accurate / Frequently (5-6 days per week)					
5	Very accurate / Always (7 days per week)					
	**Scoring**					
	Total BOS: sum all Q1-Q10					
	Domain1 (Excessive thinking): Q2+Q5+Q8					
	Domain2 (Hypersensitivity): Q3+Q6+Q9					
	Domain3 (Restless behavior): Q1+Q4+Q7+Q10					

Table S3. Cut-off scores of the HADS and BOS (n=613)

Domains	Cut-off point		Sensitivity (%)	Specificity (%)	PPV (%)	NPV (%)	Youden's index
Anxiety<br>AUC: 0.73	*>Mean+1SD*	9	0.46	0.89	0.51	0.87	0.35
	Clinical	4	0.93	0.25	0.23	0.94	0.18
	Specific	10	0.35	0.93	0.55	0.85	0.28
	Optimal	7	0.71	0.65	0.33	0.90	0.36
Depression<br>AUC: 0.65	*>Mean+1SD*	9	0.26	0.87	0.33	0.83	0.13
	Clinical	3	0.94	0.12	0.21	0.88	0.06
	Specific	10	0.16	0.92	0.35	0.82	0.09
	Optimal	7	0.55	0.72	0.32	0.87	0.27
HADS Total score<br>AUC: 0. 73	*>Mean+1SD*	16	0.49	0.84	0.43	0.87	0.33
	Clinical	9	0.91	0.32	0.25	0.93	0.23
	Specific	18	0.30	0.92	0.47	0.84	0.21
	Optimal	12	0.76	0.62	0.33	0.91	0.38
Excessive thinking<br>AUC: 0.75	*>Mean+1SD*	9	0.55	0.80	0.31	0.92	0.36
	Clinical	6	0.95	0.31	0.18	0.98	0.26
	Specific	11	0.18	0.95	0.36	0.88	0.13
	Optimal	8	0.66	0.71	0.26	0.93	0.37
Hypersensitivity<br>AUC: 0.74	*>Mean+1SD*	8	0.37	0.85	0.28	0.90	0.22
	Clinical	5	0.90	0.50	0.22	0.97	0.40
	Specific	9	0.24	0.91	0.29	0.88	0.15
	Optimal	6	0.83	0.60	0.25	0.96	0.43
Restless behavior<br>AUC: 0.69	*>Mean+1SD*	12	0.35	0.76	0.20	0.88	0.13
	Clinical	7	0.92	0.44	0.20	0.97	0.36
	Specific	14	0.16	0.91	0.21	0.87	0.06
	Optimal	8	0.87	0.50	0.21	0.96	0.37
BOS-10 Total score<br>AUC: 0.76	*>Mean+1SD*	29	0.35	0.87	0.30	0.89	0.22
	Clinical	20	0.92	0.52	0.23	0.97	0.36
	Specific	31	0.19	0.91	0.25	0.88	0.10
	Optimal	21	0.88	0.56	0.24	0.97	0.44

AUC: area under the curve. BOS-10: brain overwork scale-10. HADS: hospital anxiety and depression scale. n: count. NPV: negative predictive value. PPV: positive predictive value. SD: standard deviation.

Table S4. HADS scores by different countries.

Countries	n	Anxiety (cut-off score: 8)	Depression (cut-off score: 8)	Total (cut-off score: 17)
Mongolia (this study)	613	9.2±4.2 (34.5%)	8.2±3.7 (20%)	17.3±6.6 (20.8%)
Colombia (Hinz et al. 2014)	1500	4.6±3.6	4.3±3.9	13.0±7.8
Germany (Hinz et al. 2011)	4410	4.7±3.5 (21%)	4.7±3.9 (23%)	9.5±6.8 (30.2%)
Italy (Iani et al. 2014)	1599	7.6±4.4	5.4±4.0	13.0±7.8
Russia (Kibitov et al. 2021)	2610		4.4±2.8 (14.4%)	
UK (Breeman et al. 2015)	6189	6.2±4.1	3.9±3.7	10.1±7.9
UK (Crawford et al. 2001)	1792	6.1±3.8	3.7±3.1	9.8±6.0

HADS: hospital anxiety and depression scale. n: count.
